# Atrial fibrillation screening for stroke prevention: an instrumental variables meta-analysis addressing varying participation rates

**DOI:** 10.1093/europace/euaf030

**Published:** 2025-02-15

**Authors:** Carl Bonander, Niklas Jakobsson, Katrin Kemp Gudmundsdottir, Emma Svennberg, Johan Engdahl

**Affiliations:** School of Public Health and Community Medicine, Institute of Medicine, University of Gothenburg, Box 469, SE-405 30 Gothenburg, Sweden; Center for Societal Risk Research, Karlstad University, Universitetsgatan 2, SE-651 88 Karlstad, Sweden; Karlstad Business School, Karlstad University, Karlstad, Sweden; Department of Clinical Sciences, Karolinska Institutet, Danderyd University Hospital, Stockholm, Sweden; Department of Medicine Huddinge (MedH), Karolinska Institutet, Karolinska University Hospital, Stockholm, Sweden; Department of Clinical Sciences, Karolinska Institutet, Danderyd University Hospital, Stockholm, Sweden; Department of Cardiology, Danderyd University Hospital, Stockholm, Sweden

**Keywords:** Atrial fibrillation screening, Mass screening, Randomized controlled trial, Meta-analysis

## Introduction

Patients with atrial fibrillation (AF) face an elevated risk of ischaemic stroke. Mass screening for AF could be a cost-effective way to mitigate this risk through early detection and initiation of stroke-protective therapy.^1^ However, its effectiveness remains debated, as no trial has yet demonstrated conclusive evidence of stroke risk reduction. Meta-analytic evidence, however, suggests a modest benefit {hazard ratio [HR] = 0.91 [95% confidence interval (CI): 0.84–0.99], based on four trials published by February 2022}.^2^ In September 2024, we extended this search using the same methodology and inclusion criteria established by McIntyre *et al*.^2^ (for details, see https://osf.io/s3c98/). This update identified two new trials^3,4^ and an updated report with extended follow-up from a previously included trial,^5^ suggesting that it may be time to revisit the meta-analytic evidence.

Existing trials have employed one of two recruitment strategies: (i) randomization to screening in patients who already accepted the study invitation,^4–7^ or (ii) randomization to invitation in people initially unaware, with controls monitored solely via registers (STROKESTOP^3,8^). The first typically yields higher screening participation because participants have already consented, whereas the second often yields lower rates due to invitation non-response.

Given the absence of conclusive evidence from individual trials, meta-analyses are likely to remain essential for evaluating the effectiveness of AF screening. However, with the recent publication of STROKESTOP II^3^—one of the largest trials on the topic alongside STROKESTOP I^8^—we caution against pooling intention-to-screen estimates from these studies with those from other trials. The STROKESTOP trials evaluated the population-level impact of an AF screening invitation, with screening participation rates of 51.3%^8^ and 49.2%,^3^ respectively. In contrast, the other trials randomized participants only after recruitment, achieving near-perfect participation in their screening arms.^4–7^ Thus, while the STROKESTOP trials provide data on the effectiveness of screening invitations at the population level, the other trials more closely measure the patient-level benefits of screening participation.

Instrumental variables (IV) estimation offers a formal method to recover patient-level effects from trials with imperfect compliance while preserving the randomization’s unbiasedness. This approach, recently applied by Angrist and Hull^9^ to reconcile conflicting results in colorectal cancer screening trials with varying participation rates, can also be applied here. In this letter, we re-analyse six AF screening trials using IV estimation and demonstrate how these corrections can affect meta-analytic results.

## Methods

Our analysis includes six trials reporting stroke outcomes: four trials from a prior meta-analysis^2^ and two additional trials.^3,4^ We also incorporated extended follow-up data from one previously included trial.^5^ All trials were randomized, used electrocardiogram-based screening, and enrolled participants without prior stroke. Complete methods and literature search details are available in our Open Science Framework repository, https://osf.io/s3c98/.

We used IV analysis to address varying participation rates across trials. Conceptually, IV isolates the average effect among screening compliers by dividing the as-randomized (intention-to-screen) estimate by the proportion of screening-assigned participants who actually underwent screening, thereby avoiding the selection bias that can arise in conventional per-protocol analysis.^9^

From each trial, we extracted the participation rate, defined as the proportion of individuals assigned to screening who underwent at least one component of the AF screening process. We re-analysed incident stroke (ischaemic or haemorrhagic) outcomes with individual-level data from STROKESTOP I^8^ (*n* = 27 975; 75/76-year-olds from two Swedish regions) and II^3^ (*n* = 27 786; 75/76-year-olds from one Swedish region). Details of these trials are published elsewhere.^3,8^ For these two studies, we used Cox proportional hazards models with principal stratification weights for IV estimation.^10^ For trials with only aggregate data, we applied an approximate method that scales the log(HR) and its standard error by the screening participation rate.^9^

We then pooled HRs in two random-effects meta-analysis models with: (i) as-randomized estimates and (ii) IV-adjusted estimates. Effects are reported as HRs with 95% CIs. Individual-level IV analyses were conducted using Stata (StataCorp, 2023; Version 18.0). All other analyses were performed in R (R Core Team, 2024; Version 4.4.1).

### Ethics

Ethical approval for STROKESTOP I and II was granted by regional ethics committees (diary numbers 2011-1363-31/3 and 2015/2079–31/1, respectively).

## Results

**Figure 1:**
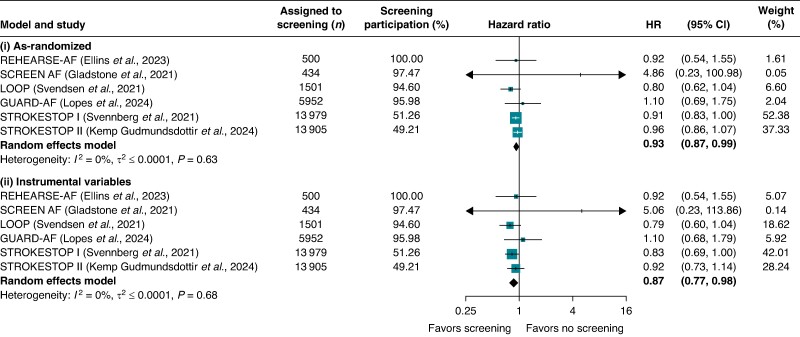
Pooled hazard ratios (HRs) from two random-effects models comparing stroke outcomes between atrial fibrillation screening and no-screening arms: (i) as-randomized and (ii) after instrumental variable adjustment for varying participation rates. Stroke outcomes were defined as follows: stroke, transient ischaemic attack, or systemic embolism (REHEARSE-AF); stroke or systemic embolism (LOOP); ischaemic or haemorrhagic stroke (GUARD-AF, STROKESTOP I, STROKESTOP II); and ischaemic stroke (SCREEN-AF). Participation was defined as undergoing at least one AF screening component: in REHEARSE-AF, participants with ≥1 ECG measurement; in SCREEN-AF, wearing the first cECG monitor; in LOOP, successful implant installation; in GUARD-AF, returning patches with analysable data; and in STROKESTOP I and II, attending the initial clinical visit.

The as-randomized meta-analysis yielded a pooled HR for stroke of 0.93 (95% CI: 0.87–0.99), favouring screening (*Figure 1*). Screening participation rates were 51.3% and 49.2% in STROKESTOP I and II, respectively, and ranged from 94.6% to 100% in the other four trials.

In STROKESTOP I, 876 strokes occurred in the screening arm and 952 in the control arm (84 907 vs. 84 320 person-years), giving an as-randomized HR of 0.91 (95% CI: 0.83–1.00) and an IV-adjusted HR of 0.83 (95% CI: 0.69–1.00). In STROKESTOP II, 636 vs. 665 strokes (68 152 vs. 68 480 person-years) yielded an as-randomized HR of 0.96 (95% CI: 0.86–1.07) and an IV-adjusted HR of 0.92 (95% CI: 0.73–1.14).

Because participation rates exceeded 94% in the remaining four trials, their as-randomized and IV-adjusted results differed only slightly (*Figure 1*). Overall, the IV meta-analysis produced a pooled HR of 0.87 (95% CI: 0.77–0.98). Sensitivity analyses using the approximate method for all studies, or leaving the other four trials unadjusted, yielded nearly identical results [HR = 0.87 (95% CI, 0.78–0.98) and HR = 0.87 (95% CI, 0.77–0.98), respectively].

## Discussion

Our study presents participation-adjusted estimates of AF screening effects from six trials and new meta-analytic estimates of how screening participation influences stroke outcomes. After accounting for varying participation rates across trials, our estimates suggest a slightly larger patient-level risk reduction than suggested by as-randomized (intention-to-screen) estimates. We encourage pragmatic screening trials to routinely report participation-adjusted estimates to strengthen future meta-analyses and inform policy and clinical decisions.

Nevertheless, some limitations warrant caution. First, although IV adjustments can address differences in participation rates, other variations among studies—such as differences in populations and screening approaches—still remain.^2^ Secondly, the validity of our IV adjustments depends on the assumption that screening invitations affect stroke risk solely through actual participation and subsequent interventions, such as anticoagulation therapy. While this assumption is untestable, it is generally considered plausible in pragmatic screening trials.^9^ Finally, all estimates arise from individuals willing to participate in such trials, who tend to be healthier and more socioeconomically advantaged.^8^ Thus, these findings may not fully represent the average patient population.

## Data Availability

Data and code to reproduce the meta-analysis are available on the study’s repository at the Open Science Framework: https://osf.io/s3c98/. Stata code and log files are also available there for the analyses requiring individual-level data from STROKESTOP I and II. For access to these data, which cannot be published openly due to ethical and legal restrictions, please refer to the original papers and contact the original authors.
